# Dihydroartemisinin Sensitizes Lung Cancer Cells to Cisplatin Treatment by Upregulating ZIP14 Expression and Inducing Ferroptosis

**DOI:** 10.1002/cam4.70271

**Published:** 2024-10-12

**Authors:** Zhuoying Yang, Zehao Zhou, Qingyu Meng, Zhijie Chen, Liang Yun, Jianjun Jiang, Yujing He, Meijuan Dian, Ruihao Zhang, Haotian Ge, Tianbao Yan, Biying Men, Zichao Li, Xu Wu, Junming He, Shuan Rao

**Affiliations:** ^1^ Department of Thoracic Surgery, Nanfang Hospital Southern Medical University Guangzhou Guangdong China; ^2^ Department of Radiation Oncology, Peking Union Medical College Hospital Chinese Academy of Medical Sciences & Peking Union Medical College Beijing China; ^3^ Department of Hepatobiliary Surgery The Second Affiliated Hospital of Guangzhou University of Chinese Medicine Guangzhou Guangdong China

**Keywords:** cisplatin, dihydroartemisinin, ferroptosis, lung cancer, non‐transferrin iron transport, ZIP14

## Abstract

**Background:**

Despite significant advances in lung cancer treatment, cisplatin (DDP)‐based chemotherapy remains a cornerstone for managing the disease. However, the prevalence of chemoresistance presents a major challenge, limiting its effectiveness and contributing to poor outcomes. This underscores the urgent need for novel therapeutic strategies to overcome chemoresistance and improve chemotherapy efficacy in lung cancer patients. Exploring approaches to sensitize tumors to cisplatin could enhance treatment responses and overall survival rates.

**Methods and Results:**

Our study utilized a variety of lung cancer models, including cell lines, mouse models, and patient‐derived organoids, to validate the synergistic cytotoxic effects of dihydroartemisinin (DHA) and cisplatin (DDP). When combined with DDP, we demonstrate that DHA is a promising therapeutic agent that effectively triggers ferroptosis in lung cancer cells, offering a potential strategy for overcoming chemoresistance. Mechanistically, the combination of DHA and DDP synergistically enhances ZIP14 expression, modulating iron homeostasis and upregulating oxidative stress, leading to both in vitro and in vivo ferroptosis. Notably, our findings revealed that the sequential administration of DDP followed by DHA significantly increases ZIP14 expression and induces superior therapeutic outcomes compared to the simultaneous administration or DHA followed by DDP. This observation underscores the importance of the drug administration order in optimizing treatment efficacy, providing new insights into enhancing chemotherapy response in lung cancer.

**Conclusion:**

Our findings suggest that combining dihydroartemisinin (DHA) with cisplatin (DDP) presents a promising strategy to overcome chemoresistance in lung cancer patients. Importantly, administering DHA during chemotherapy intervals could further optimize treatment outcomes, enhancing the overall efficacy of lung cancer chemotherapy.

## Graphical abstract

Dihydroartemisinin combined with Cisplatin increase the intracellular ferrous iron content by upregulating ZIP14 expression, disrupting cellular oxidative stress, and enhancing the sensitivity of lung cancer cells to ferroptosis. Moreover, sequential treatment of DDP then DHA offers superior therapeutic outcomes.

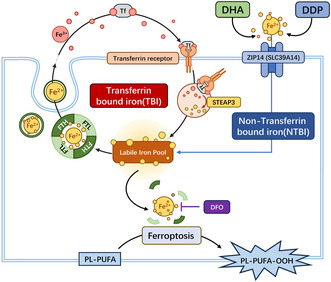

**Abbreviations**
CCK‐8: cell counting Kit‐8
DCFH‐DA: 2′,7′‐dichlorofluorescin diacetate
DDP: cisplatin
DFO: deferoxamine mesylate
DHA: dihydroartemisinin
MDA: malondialdehyde
Necro: necrosulfonamide
ZVAD: Z‐VAD‐FMK

## 1 Introduction

Lung cancer ranks as the most commonly diagnosed cancer and a leading cause of cancer‐related deaths worldwide [1, 2]. Despite notable progress in diagnostic and therapeutic approaches, such as molecular targeted therapy and immunotherapy, lung cancer remains highly lethal, primarily due to its resistance to anticancer treatments and disease progression [3, 4]. Chemotherapy remains a cornerstone in the management of lung cancer, particularly for patients who show no response to targeted or immunotherapy, as well as those in advanced stages with distant metastasis [2, 5]. However, the clinical application of chemotherapy is hampered by its severe side effects, often resulting in treatment discontinuation and the development of acquired drug resistance [6, 7]. Hence, there is an urgent need to develop novel therapeutic strategies that are both more effective and less toxic, with the ability to sensitize cancer cells to chemotherapy agents [8].

Ferroptosis, a regulated form of cell death driven by reactive oxygen species (ROS) and iron accumulation [9, 10, 11], presents an intriguing avenue for overcoming resistance mechanisms in lung cancer cells, thereby offering a novel therapeutic approach [12, 13]. The regulation of ferroptosis primarily relies on TFRC‐mediated transferrin‐bound iron (TBI) transport of ferrous ions. However, under certain circumstances, the ZIP14‐mediated non‐transferrin‐bound iron (NTBI) can also play an important role, leading to increased intracellular iron levels and subsequent ROS generation. Therefore, modulating ZIP14 expression may play a critical role in enhancing ferroptosis, particularly in the context of chemotherapy [14, 15].

Drug repurposing, which involves identifying new applications for approved or clinically tested drugs, offers an efficient strategy that reduces development timelines and costs while leveraging the established safety profiles of existing medications [16, 17]. Dihydroartemisinin (DHA), derived from the traditional Chinese medicine *Artemisia annua*, has primarily served as an antimalarial agent and has shown promising antitumor potential in various cancers, including lung cancer [18, 19, 20]. Importantly, previous reports have implied the synergistic antitumor effect of DHA in combination with cisplatin (DDP) in the treatment of various cancer [21, 22, 23]. This study aims to investigate whether DHA could enhance the sensitivity of lung cancer cells to DDP therapy, and we seek to elucidate the detailed mechanisms, specifically how DHA synergizes with DDP to induce the cell death of lung cancer cells.

## 2 Results

### 2.1 DHA Demonstrates a Synergistic Cytotoxic Effect With DDP Across Various Lung Cancer Models

**FIGURE 1:**
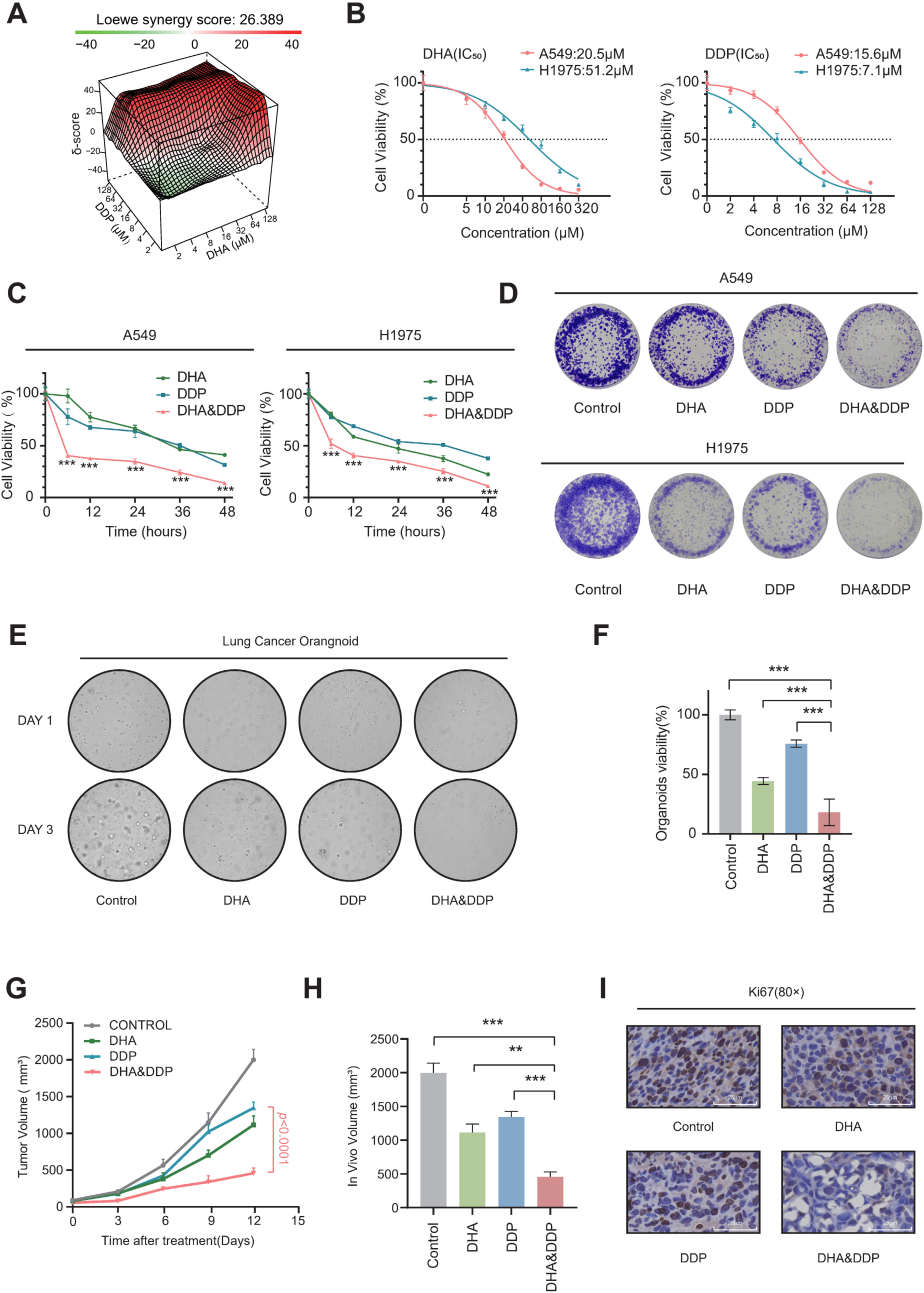
Synergistic antitumor effect of DHA in combination with DDP. (A) 3D Finder Synergy map illustrating Loewe synergy scores between DHA and DDP score < −10, antagonistic effect, −10 < score < 10, additive effect, 10 > score, synergistic effect. (B) IC_50_ values for A549 or H1975 cells treated with DHA or DDP, mean ± SD, *n* = 6. (C) A549 or H1975 cells were incubated with DHA (20 μΜ for A549 or 40 μΜ for H1975) and DDP (10 μΜ) for 48 h, and cell viability was subsequently measured at different time points using the CCK‐8 assay kit, mean ± SD, *n* = 6. (D) The colony formation assay of A549 and H1975 cells was conducted under condition of either mono or combined treatments with DHA (20 μΜ for A549 or 40 μΜ for H1975) and DDP (10 μΜ) for 5 days. (E) Representative images of human lung cancer organoids after treatment with DHA (50 μM) and DDP (15 μM), either alone or in combination for 72 h. Upper panel: Before treatment (40×), lower panel: After treatment (40×). (F) Lung cancer organoids were treated with DHA (50 μM) and DDP (15 μM) for 72 h, and their viability was assessed using ATP assay. Mean ± SD, *n* = 6. (G) Xenografts of Lewis lung cancer cells were established in C57BL/6 mice and treated with control (PBS), DHA (20 mg/kg), DDP (10 mg/kg), DHA (20 mg/kg), and DDP (10 mg/kg), respectively. Tumor volume was measured every 3 days, mean ± SEM, *n* = 5. (H) Tumor volume analysis of (G) on day 12, mean ± SEM, *n* = 5. (I) Representative Ki67 staining of subcutaneous tumor samples from the control, DHA, DDP, and combination treated groups of (G). Scale bars: 25 μm. *p < 0.05, **p < 0.01, and ***p < 0.001.

Utilizing the online tool 3D Finder Synergy, we calculated Loewe synergy scores to assess the synergistic potential of different drug combinations [24]. The combination of DHA and DDP exhibited a significantly high Loewe synergy score of 26.389, indicating a potent antitumor effect (Figure 1A). To evaluate the toxic effects of DHA and DDP, we initially determined the half‐maximal inhibitory concentration (IC_50_) values for DHA and DDP in A549 and H1975 cells, respectively. Both compounds inhibited lung cancer cell growth in a dose‐dependent manner (Figure 1B). Interestingly, the combination of DHA and DDP significantly suppressed lung cancer cell growth (Figure 1C) and colony formation (Figure 1D) compared to monotherapy with DHA or DDP alone. Furthermore, the combined treatment of DHA and DDP demonstrated superior antitumor effects in human lung cancer organoids (Figure 1E,F). To investigate the therapeutic efficacy of the DHA and DDP combination in vivo, we established a subcutaneous tumor model in mice bearing Lewis lung cancer cells. The mice were randomly divided into four groups and treated with vehicle control, DHA, DDP, or the combination of DHA and DDP. Consistently, the tumor‐suppressing effect was more pronounced in the DHA and DDP combination‐treated group compared to DHA or DDP monotherapy group, with no observed toxic effects (Figure 1G–I). These findings suggest that DHA exhibits a synergistic cytotoxic effect with DDP across diverse lung cancer models.

### 2.2 Combination of DHA and DDP Induces Ferroptosis in Lung Cancer Cells

**FIGURE 2:**
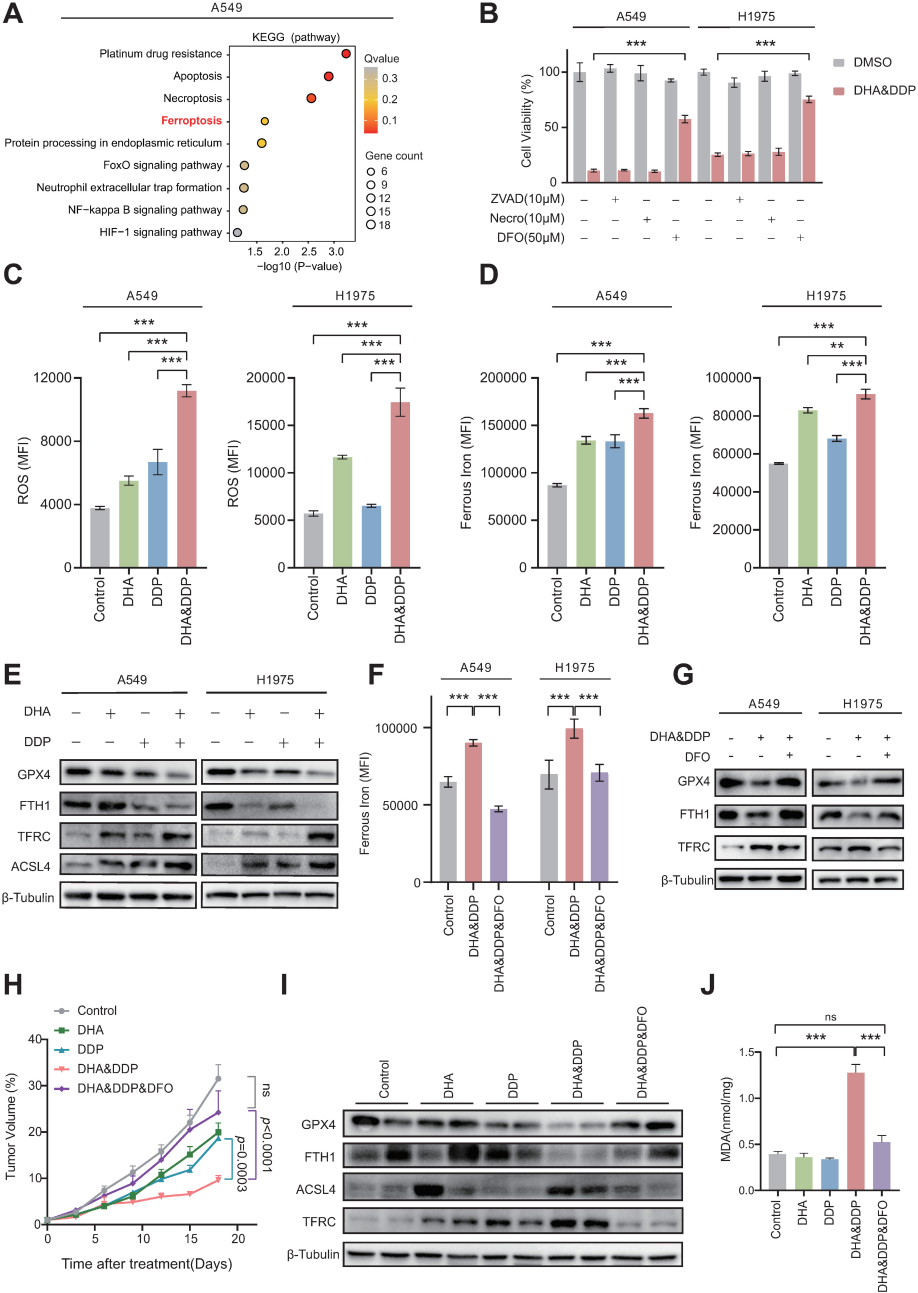
DHA co‐treatment with DDP synergistically activates ferroptosis and can be rescued by DFO. (A) Bubble plot depicting the Kyoto Encyclopedia of Genes and Genomes (KEGG) pathway enrichment of Differentially Expressed Genes (DEGs) in A549 cells treated with both DHA (20 μΜ) and DDP (10 μΜ). (B) A549 or H1975 cells were incubated with DHA (20 μM for A549 or 40 μM for H1975) and DDP (10 μM) for 48 h, with various cell death inhibitors added simultaneously to assess their effects on cell viability. Mean ± SD, *n* = 6. (C) The mean fluorescence intensity of intracellular ROS in A549 and H1975 cells treated with DHA (20 μΜ for A549 or 40 μΜ for H1975) and DDP (10 μΜ) alone or in combination for 48 h. Mean ± SD, *n* = 3. (D) The mean fluorescence intensity of intracellular ferrous iron in A549 and H1975 cells treated with DHA (20 μΜ for A549 or 40 μΜ for H1975) and DDP (10 μΜ) alone or in combination for 48 h. Mean ± SD, *n* = 3. (E) Western blot was used to detect the expression of the ferroptosis‐associated proteins in A549 and H1975 cells treated with DHA (20 μΜ for A549 or 40 μΜ for H1975) and DDP (10 μΜ) alone or in combination for 48 h. (F) Flow cytometry was used to evaluate intracellular ferrous iron levels in A549 and H1975 cells treated with DHA (20 μΜ for A549 or 40 μΜ for H1975) and DDP (10 μΜ) with or without DFO (50 μΜ) for 48 h, cells were pretreated with DFO (50 μΜ) for 12 h, Mean ± SD, *n* = 3. (G) Western blot was used to detect the expression of the ferroptosis‐associated proteins in A549 and H1975 cells treated with DHA (20 μΜ for A549 or 40 μΜ for H1975) and DDP (10 μΜ) with or without DFO (50 μΜ) for 48 h, cells were pretreated with DFO (50 μΜ) for 12 h. (H) Xenografts of Lewis lung cancer cells were established in C57BL/6 mice and treated with Control (PBS), DHA (20 mg/kg), DDP (10 mg/kg), DHA (20 mg/kg) and DDP (10 mg/kg), and DHA and DDP plus DFO (100 mg/kg). Tumor volume was measured every 3 days, mean ± SEM, *n* = 6. (I) Western blot was used to detect the ferroptosis‐associated proteins on lysates isolated from xenografts in (H). (J) The content of MDA in tumor tissue homogenates of mice in (H), presented as mean ± SD, *n* = 3. *p < 0.05, **p < 0.01, ***p < 0.001, ns, statistically not significant.

To understand the molecular basis of how DHA and DDP synergistically kill tumor cells, we performed a transcriptome analysis which revealed genes annotated to apoptosis, necroptosis as well as ferroptosis were enriched in the combined drug‐treated A549 cells (Figure 2A). By using different small molecular inhibitors, including Z‐VAD‐FMK (Z‐VAD, apoptosis inhibitor) [25], Necrosulfonamide (Necro, necroptosis inhibitor) [26], and Deferoxamine (DFO, iron‐chelating agent and ferroptosis inhibitor) [27], we proved that ferroptosis was the major cell death subtype induced by DHA and DDP (Figure 2B), this finding was further supported by the gene set enrichment analysis (GSEA) with our RNA‐seq data (Figure S1A). We then evaluated ferroptosis level in cells with different treatments by measuring ROS and ferrous, which indicated that the DHA and DDP combination significantly increased both ROS and ferrous iron levels in lung cancer cells (Figures 2C,D and S1B); however, either apoptosis or autophagy was not triggered by DHA and DDP treatment (Figure S1E,F). Subsequently, we determined protein level of key ferroptosis indicators, including GPX4, FTH1, TFRC, and ACSL4 by performing western blot, suggesting ferroptosis was indeed activated in DHA and DDP‐treated cells (Figure 2E). Importantly, by pretreating cells with ferroptosis inhibitor DFO for 12 h, we showed that DFO effectively reversed various ferroptotic phenotypes induced by DHA and DDP co‐treatment in lung cancer cells (Figures 2F,G and S1C,D). Furthermore, DFO administration significantly diminished the tumor‐suppressing effects of DHA and DDP in vivo by inhibiting ferroptosis (Figures 2H–J and S1G,H).

### 2.3 The Transferrin Receptor Binding Iron (TBI) Pathway May Not Be the Primary Downstream Pathway Responsible for Ferrous Accumulation Induced by Combined DHA and DDP Treatment

**FIGURE 3:**
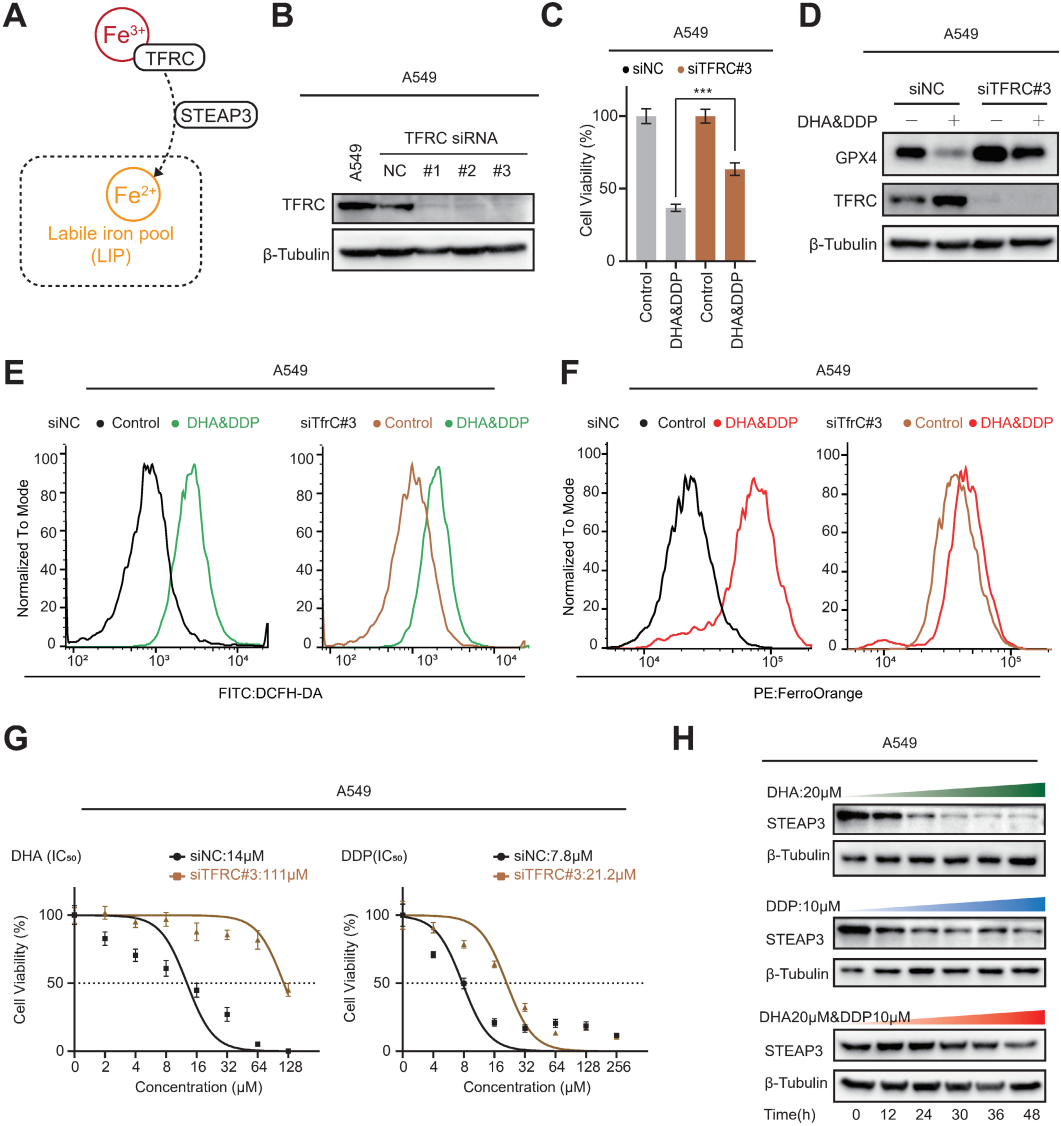
Reduced TFRC expression promotes tolerance to DHA&DDP‐induced ferroptosis. (A) Schematic diagram showing how the transferrin receptor intakes iron from extracellular environment. (B) Western blot was used to verify the efficiency of TFRC siRNA knockdown in A549 cells. (C) The viability evaluation of A549 cells treated with DHA (20 μM) and DDP (10 μM) for 48 h after TFRC knockdown by CCK‐8 assay, mean ± SD, *n* = 3. (D) Western blot was used to detect the expression of GPX4 protein in A549 cells treated with DHA (20 μM) and DDP (10 μM) for 48 h after TFRC knockdown. (E,F) Flow cytometry was used to evaluate the changes in ROS (left) and ferrous iron (right) levels in A549 cells treated with DHA (20 μM) and DDP (10 μM) for 48 h after TFRC knockdown. (G) IC_50_ values of A549 and H1975 cells treated with DHA or DDP for 48 h after TFRC knockdown. (H) Western blot was used to detect STEAP3 protein expression in A549 cells treated with mono or combined treatment of DHA (20 μM) and DDP (10 μM) for different time points. ***p < 0.001.

To investigate how combined DHA and DDP treatment induces ferroptosis in lung cancer cells, we first assessed alterations in the Transferrin receptor binding iron (TBI) pathway which was mediated by Transferrin Receptor Proteins (TFRC) and STEAP3 (Figure 3A). TFRC, a cell surface membrane protein, plays a crucial role in transporting trivalent iron ions [28, 29]. Using siRNA technology, we successfully knocked down TFRC in lung cancer cells (Figure 3B), which led to a significant restoration of cell viability and GPX4 expression in TFRC knocked down cells treated with combined DHA and DDP (Figure 3C,D). Furthermore, inhibition of TFRC attenuated ROS and ferrous iron accumulation induced by DHA and DDP co‐treatment (Figure 3E,F), and TFRC deficiency also increased the resistance of lung cancer cells to DHA or DDP treatment, respectively (Figure 3G). Molecular docking is a computational technique used to predict the binding affinity and binding mode of receptor–drug molecular complexes [30, 31], our molecular docking data suggested the potential of DHA to bind with TFRC (Figure S2A).

Interestingly, despite ferrous accumulation in cells, STEAP3, the key enzyme in the TBI transport pathway which is responsible for converting trivalent to divalent iron within cells [32, 33], was significantly downregulated in either DHA or DDP mono‐treated cells, as well as in cells treated with combined DHA and DDP (Figure 3H). These findings suggest that while ferrous accumulation occurs, the TFRC/STEAP3‐mediated TBI pathway may not be the primary downstream pathway responsible for the ferrous accumulation induced by combined DHA and DDP treatment.

### 2.4 ZIP14 is the Pivotal Target to Mediate Synergistic Tumor Suppressing Effects Induced by DHA and DDP

**FIGURE 4:**
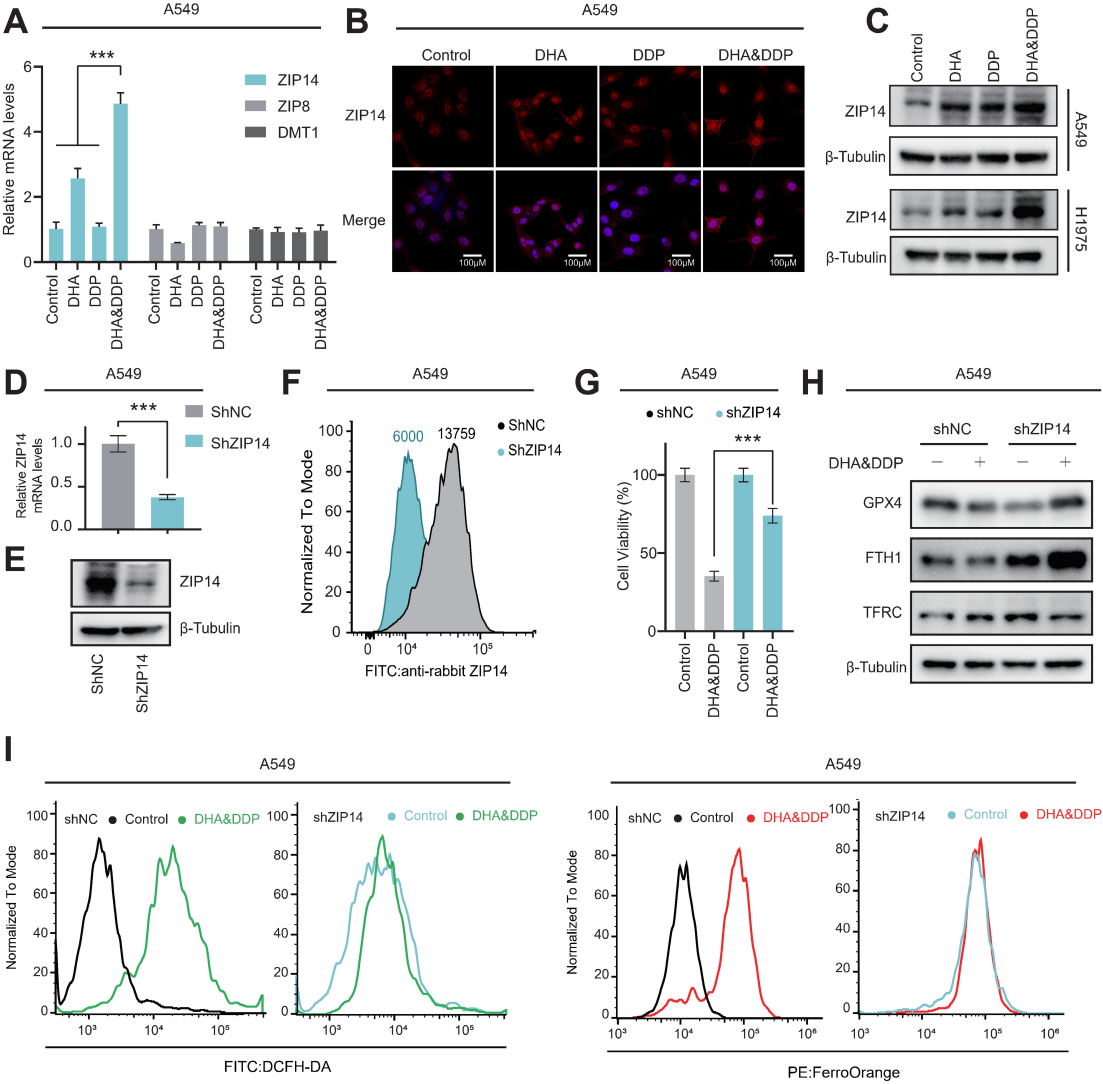
Decreased expression of ZIP14 contributed to the tolerance of DHA&DDP‐induced ferroptosis. (A) The qPCR analysis of ZIP14, ZIP8, and DMT1 levels in A549 cells treated with DHA (20 μM) and DDP (10 μM) alone or in combination for 48 h, mean ± SD, *n* = 3. (B) Immunofluorescence imaging of ZIP14 (red) and DAPI (blue) under mono or combined treatment of DHA (20 μΜ) and DDP (10 μΜ) in A549 cells for 48 h. Scale bars: 100 μm. (C) Western blot was used to detect the expression of ZIP14 proteins in A549 and H1975 cells treated with DHA (20 μΜ for A549 or 40 μΜ for H1975) and DDP (10 μΜ) alone or in combination for 48 h. (D) The level of ZIP14 mRNA was measured by qPCR after RNA interference using shRNA in A549 cells, mean ± SD, *n* = 3. (E) Western blot was used to detect the expression ZIP14 proteins in A549 cells treated with shZIP14. (F) Membrane‐associated ZIP14 protein levels in shNC or shZIP14 A549 cells were measured using flow cytometry. (G) The viability of shNC or shZIP14 A549 cells treated with DHA (20 μM) and DDP (10 μM) for 48 h by CCK‐8 assay, mean ± SD, *n* = 3. (H) Western blot was used to detect the expression of the ferroptosis‐associated proteins in shNC or shZIP14 A549 cells treated with DHA (20 μM) and DDP (10 μM) for 48 h. (I) Flow cytometry was used to evaluate changes in ROS (left) and ferrous iron (right) levels in shNC or shZIP14 A549 cells treated with DHA (20 μM) and DDP (10 μM) for 48 h. ***p < 0.001.

In our quest to explore other targets involved in the synergistic effect of DHA and DDP, we assessed the transcriptional changes of key divalent metal transporter genes [13, 34]. Among them, only ZIP14 exhibited a noticeable upregulation in cells treated with both DHA and DDP, while ZIP8 or DMT1 showed no significant changes (Figure 4A). Immunofluorescence staining further confirmed the enhanced expression of ZIP14 in cells treated with DHA and DDP (Figure 4B). In addition, molecular docking experiments suggested that DHA had a strong binding potential with ZIP14 (Figure S2B), and we further confirmed that DHA could directly bind to the ZIP14 protein using the Cellular Thermal Shift Assay (CETSA) (Figure S2C). To validate the mechanistic role of ZIP14, we established stable ZIP14 knocked down (shZIP14) cells in A549 and H1975 cells. qPCR and FACs analysis demonstrated robust knockdown efficiency of ZIP14 in shZIP14 cells (Figures 4D–F and S2D–F). Subsequent investigations revealed that inhibition of ZIP14 markedly reduced cellular sensitivity to the concomitant administration of DHA and DDP (Figures 4G and S2G) by impeding ferroptosis (Figures 4H and S2H). Furthermore, the augmentation of ROS and ferrous iron levels in control cells (shNC) induced by DHA and DDP treatment was completely abolished in ZIP14‐deficient cells (Figures 4I and S2I), indicating that ZIP14 is indeed the pivotal target of the DHA and DDP combination.

### 2.5 Sequential Administration of DDP and DHA Exerts Superior Antitumor Effect

**FIGURE 5:**
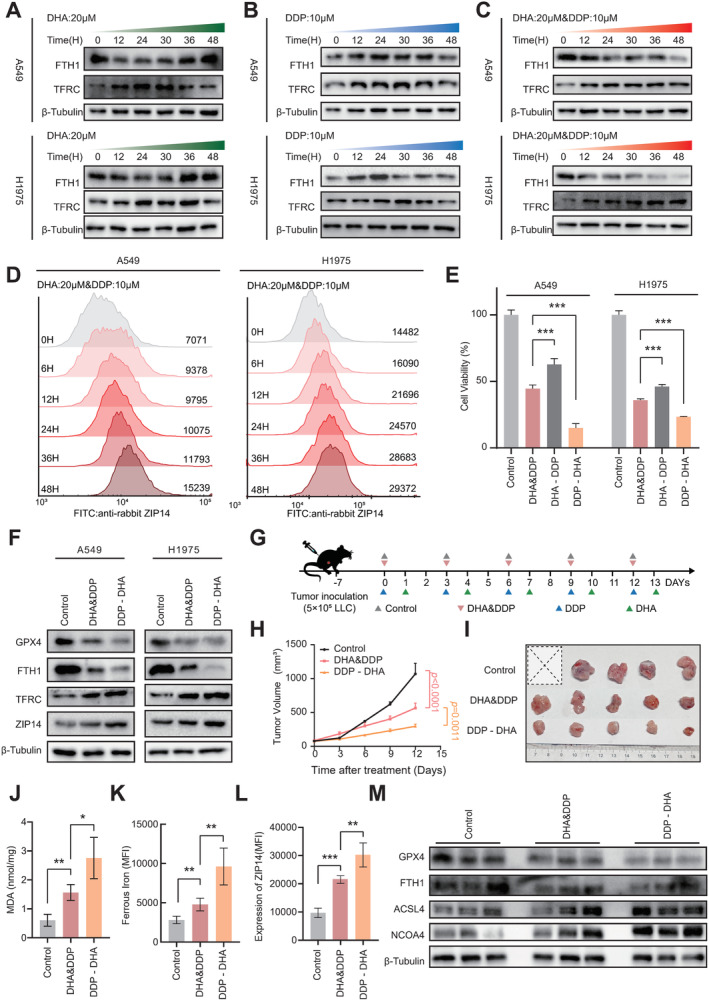
Sequential administration of DDP and DHA has therapeutic differences. (A–C) Western blot was used to detect FTH1 and TFRC expression in A549 and H1975 cells treated with DHA (20 μΜ) and DDP (10 μΜ) alone or in combination at different time points. (D) Flow cytometry was used to detect the expression of ZIP14 in A549 and H1975 cells treated with DHA (20 μM) and DDP (10 μM) at different time points. (E) Cell viability analysis of A549 and H1975 cells with different treatment combinations of DHA (20 μM) and DDP (10 μM) as depicted in (Figure S3B), mean ± SD, *n* = 6. (F) Western blot was used to detect the expression of the ferroptosis‐associated proteins in A549 and H1975 cells treated with co‐treatment or sequential treatment of DHA (20 μΜ for A549 or 40 μΜ for H1975) and DDP (10 μΜ). (G) The schematic diagram illustrates drug administration and experimental design for in vivo Lewis lung cancer model. (H) Xenografts of Lewis lung cancer cells were established in C57BL/6 mice and treated with Control (PBS), DHA (20 mg/kg) and DDP (10 mg/kg) simultaneously, or DHA (20 mg/kg) and DDP (10 mg/kg) sequentially. Tumor volume was measured every 3 days and presented as mean ± SEM, *n* = 5. (I) Representative image of tumor samples in (G) with indicated treatment. (J) Evaluation of MDA in tumor tissue homogenates of mice in (G) with indicated treatment, mean ± SD, *n* = 3. (K,L) Flow cytometry was used to evaluate the level of ferrous iron and expression of ZIP14 in mice tumor tissues with indicated treatment in (G), mean ± SD, *n* = 3. (M) The expression of ferroptosis‐related proteins in tumor tissues of mice in (G) with indicated treatment was detected by western blot. *p < 0.05, **p < 0.01, and ***p < 0.005.

As ferroptosis is activated by DDP and DHA co‐treatment, we then queried how this cell death pathway regulated in a time‐dependent manner. Interestingly, either DHA or DDP alone could affect FTH1 and TFRC expression shortly (Figure 5A,B), but these changes were disappeared when the exposure time increased. In contrast, when cells were co‐treated with DHA and DDP, we observed constant ferroptosis activation as indicated by FTH1 and TFRC expression (Figure 5C); furthermore, ZIP14 expression was stably increased as the treatment time prolonged (Figures 5D and S3A), suggesting the antitumor effect of combined DDP and DHA was mediated by increasing ZIP14 expression.

When we examined the expression of FTH1, we noticed DHA and DDP had opposite effect on FTH1 expression (Figure 5A,B), suggesting their effect to ferritin export might be compromised which might affect the ferroptosis activation. In order to optimize the therapeutic effect, we designed multiple dosing regimens by co‐application of DHA and DDP (named DHA&DDP hereafter), sequential application of DHA and DDP (named DHA‐DDP hereafter) or DDP and DHA (named DDP‐DHA hereafter) (Figure S3B). Surprisingly, the DHA‐DDP treatment had a mild tumor cell killing effect, while the DDP‐DHA treatment achieved the best antitumor effect compared to other treatments. Indeed, the DDP‐DHA treatment induced more profound ferroptosis as evidenced by lower expression of GPX4 and FTH1, as well as higher expression of TFRC and ZIP14 (Figures 5F and S3C,D). The levels of ferrous iron and ROS also confirmed that ferroptosis activation was more significant in DDP‐DHA‐treated cells (Figure S3E,F). We then designed an in vivo experiment to evaluate the therapeutic outcome of DHA&DDP and DDP‐DHA (DDP was administrated first and then DHA was given with 24 h interval every 3 days) (Figure 5G). In line with the cell viability assay, DDP‐DHA treatment dramatically suppressed tumor progression (Figure 5H) and tumor burden (Figure 5I) in mice as compared to DHA&DDP‐treated mice. Moreover, we evaluated the level of MDA, ferrous iron, and ZIP14 expression in tumor tissues; all these indicators were significantly higher in DDP‐DHA‐treated mice (Figure 5J–L); in addition, other ferroptosis markers, including GPX4, FTH1, ACSL4, and NCOA4 had more significant changes in DDP‐DHA‐treated mice (Figure 5M), all these results demonstrate that sequential administration of DDP and DHA can achieve superior anticancer effects by triggering more ferroptosis.

## 3 Discussion

Chemotherapy remains an indispensable treatment option for many lung cancer patients, especially when other therapies such as immunotherapy or targeted therapy fail [35], despite the inevitable challenges of chemoresistance and severe side effects [36]. A key challenge is to develop more effective strategies to enhance chemosensitivity, such as modulating DNA damage response and drug up‐taking, or targeting important cell proliferation pathways [37, 38, 39]. Recently, many studies have revealed the role of ferroptosis in regulating the response and resistance to various chemotherapies. For example, doxorubicin can downregulate GPX4, leading to mitochondrial‐dependent ferroptosis in cardiomyocytes [40]; etoposide induces cellular metabolic reprogramming, leading to lactate accumulation in the microenvironment and causing ferroptosis resistance in non‐small cell lung cancer [41]; inhibition of METTL17 in colorectal cancer cells disrupts mitochondrial function and energy metabolism, thereby enhancing intracellular and mitochondrial lipid peroxidation and ROS levels during ferroptosis stress [42].

Previous studies have shown that the combination of DHA and DDP can induce TFRC expression, augment the TFRC‐mediated non‐transferrin‐bound iron (NTBI) uptake pathway and facilitate ferroptosis in pancreatic ductal adenocarcinoma [21]. However, our study discovered that DHA significantly enhances cisplatin's antitumor effects in lung cancer cells through the ZIP14‐mediated non‐transferrin‐bound iron (NTBI) pathway, which was different from earlier findings that primarily focus on TFRC‐mediated iron uptake [32]. Our study demonstrated that the combination of DHA and DDP, synergistically increases the expression of ZIP14, thereby elevating intracellular free iron levels, disrupting oxidative homeostasis, and ultimately inducing ferroptosis in lung cancer cells, highlighting the potential of ZIP14 as a candidate to overcome chemoresistance by regulating ferroptosis.

Another intriguing aspect of our findings is that the sequence of drug administration influences therapeutic efficacy. Specifically, sequential administration of DDP followed by DHA resulted in more profound tumor killing effects compared to concurrent use of these agents. Similar observation is also reported in a study which indicates that sequential treatment of histone deacetylase inhibitor valproic acid with etoposide can enhance drug efficacy in killing melanoma cells [43].

Many studies have shown that artemisinin derivatives combined with chemotherapy can induce various cell death pathways. For example, artesunate combined with cisplatin can affects the expression of such as Bcl‐2, Bax, and Caspase3/7, and synergistically regulates the activity of the P38/JNL/ERK1/MAPK pathway [23]; artemisinin B can activate the MAPK pathway and enhance the efficacy of cisplatin by increasing the expression of Cx43 and elevating intracellular Fe^2+^ levels in both normal and DDP‐resistant NSCLC cells [44]. Additionally, artesunate can kill cisplatin‐resistant cells by inducing G0/G1 phase arrest, regulating cell cycle regulatory proteins' expression and causing DNA damage [45]. Our research underscores that DHA and DDP can promote ferroptosis through ZIP14‐mediated regulation of iron metabolism and oxidative stress. Of note, chemotherapy resistance mechanisms are complex and diverse, including reduced drug uptake and increased drug efflux, enhanced DNA damage repair, and apoptotic defects [9, 46, 47], whether combined treatment with DHA and DDP can overcome chemoresistance by regulating these processes requires further investigation.

The clinical implication of our study is that for lung cancer patients undergoing cisplatin‐based chemotherapy, dihydroartemisinin might be a cost‐effective and efficient supplement to improve chemotherapy outcomes by inducing ferroptosis in tumor cells. Our study provides a theoretical basis for repurposing dihydroartemisinin as a potential drug candidate for lung cancer treatment.

## 4 Materials and Methods

### 4.1 Cell Lines

The human NSCLC cell lines A549 and H1975, as well as the Lewis mouse lung cancer cell line, were all obtained from China Center for Type Culture Collection (CCTCC). These cell lines were maintained in RPMI 1640 media supplemented with 10% fetal bovine serum, 100 U/mL penicillin, and 100 μg/mL streptomycin. All cells were confirmed to be free of mycoplasma contamination. Additionally, all cell cultures were maintained at 37°C in a humidified incubator under a 5% CO2 atmosphere.

### 4.2 Reagents and Antibodies

Dihydroartemisinin (> 98%) and Cisplatin (> 98%) were purchased from Macklin (Macklin, China). Z‐VAD‐FMK (HY‐16658B), Necrosulfonamide (HY‐100573), and Deferoxamine mesylate (HY‐B0988) were purchased from MCE (Med Chem Express, Princeton, USA). Antibody against GPX4 (ab125066), STEAP3 (ab151566), and ACSL4 (ab155282) were obtained from Abcam. Antibody against FTH1 (sc‐376,594) and NCOA4 (sc‐373,739) were obtained from Santa Cruz (Santa Cruz Biotechnology, Texas, USA). Antibody against TFRC (Cell Signaling, #13113S), LC3B (Cell Signaling, # 2775S), and P62 (Cell Signaling, # 5114S) were obtained from Cell Signaling (Cell Signaling Technology, Massachusetts, USA). Antibody against ZIP14 (Affinity, DF14224) was obtained from Affinity (Affinity Biosciences, Jiangsu, China) for flow cytometry and immunofluorescence, while the antibody against ZIP14 (Abclonal, A10413) was obtained from Abclonal (Wuhan, China) for western blot.

### 4.3 Cell Viability Assays

The A549 and H1975 cells were seeded in a 96‐well plate at a density of 4000 cells per well. Following drug treatment, the original culture medium was replaced, and 100 μL of medium containing 10% Cell Counting Kit‐8 (NCM biotech, Jiangsu, China) reagent was added to each well. Subsequently, the cells were incubated in a light‐avoidant environment at 37°C for 1 h. The absorbance at 450 nm was then measured for each well using a microplate reader, with the blank control group as the reference, enabling the calculation of relative cell viability and relative inhibition rate. Finally, GraphPad Prism (version 8.0) was used to determine the half‐maximal inhibitory concentration (IC_50_) values for each drug group.

### 4.4 Lung Cancer Organoid Culture and Viability Assay

Fresh lung cancer tissue from patients was washed, centrifuged, digested, and subjected to red blood cell lysis. Immune cells and fibroblasts were removed. The cells were resuspended in Matrigel, and 10 μL was seeded into a 48‐well plate. After solidifying in a 37°C incubator, additional primary medium was added, and incubation was continued. A 3D Cell Viability Assay Kit (G9681, Promega, Beijing, China) to detect organoid viability was used. Please refer to the literature for its detailed working principle [48].

### 4.5 Colony Formation Assays

For colony formation assay with monolayer culture, cells (1 × 10^4^/well) were plated in a 6‐well plate for 1 week until colonies were obviously formed. After cells were treated with DHA and (or) DDP for 5 days, the plate was fixed with methanol and gently washed and stained with crystal violet for 10 min, and then the colonies were imaged and counted.

### 4.6 Measurement of Reactive Oxygen Species

2′,7′‐Dichlorofluorescin diacetate (DCFH‐DA) (Dojindo, Kumamoto, Japan) was used to detect intracellular ROS levels. Initially, dilute DCFH‐DA in a serum‐free culture medium at a ratio of 1:1000 to achieve a final concentration of 10 μmol/L. Subsequently, after drug treatment, the original culture medium from A549 and H1975 cells was removed, an appropriate volume of diluted DCFH‐DA was added, and incubated in a light‐avoidant environment at 37°C for 30 min. Next, the cells were washed three times with cold PBS to ensure complete removal of the fluorescent probe that did not enter the cells. Finally, following the manufacturer's instructions, the fluorescence intensity in the FITC channel was measured using a flow cytometer.

### 4.7 Measurement of Ferrous Iron

To evaluate intracellular ferrous iron levels in NSCLC cells post‐drug treatment, the FerroOrange fluorescent probe (Dojindo, Kumamoto, Japan) was employed. After three times wash with PBS, the cells were incubated with a 1 μM FerroOrange working solution in a 37°C, 5% CO_2_ incubator for 30 min. Subsequently, fluorescence intensity in the PE channel was detected using a flow cytometer in accordance with the manufacturer's instructions.

### 4.8 Malondialdehyde (MDA) Assays

For MDA determination, RIPA tissue lysis buffer (Beyotime Biotechnology, Jiangsu, China) was used to extract protein from the lysed tumor tissue for obtaining protein supernatant. The working solution was prepared according to the instructions of the Lipid Peroxidation MDA Assay Kit (Beyotime Biotechnology, Jiangsu, China), and after thorough mixing, the absorbance was measured at 532 nm using a microplate reader. Following the calculation of MDA content in the sample solution, protein levels were measured using the Micro BCA Protein Assay Kit (Beyotime Biotechnology, Jiangsu, China), and expressed the MDA content per unit of protein concentration in the sample.

### 4.9 Western Blot

Total cell lysates were extracted in RIPA buffer (Beyotime Biotechnology, Jiangsu, China) on ice for 10 min. The protein concentration was quantified using the BCA Protein Assay Kit (Beyotime Biotechnology, Jiangsu, China). Subsequently, equal amounts of proteins were separated by 8%, 10%, and 12% SDS‐PAGE and transferred to PVDF membranes. The membranes were blocked with 5% nonfat milk for 1 h at room temperature and incubated with the primary antibodies at 4°C overnight. Afterward, the membranes were incubated with secondary antibodies at room temperature for 1 h and then washed three times with TBST. The protein bands were visualized using an ECL‐Plus chemiluminescence detection kit.

### 4.10 Cellular Thermal Shift Assay (CETSA)–Western Blot (WB)

The soluble protein lysate from A549 and H1975 cells was aliquoted into PCR tubes and treated with DHA (200 μmol/L) or DMSO for 1 h at room temperature before undergoing CETSA heat pulse. The samples were then heated at the indicated temperatures (35°C–60°C) for 5 min, followed by cooling at 4°C for 3 min in a Thermom Scientific (Thermo Fisher Scientific, USA). After centrifugation at 12,000 rpm for 10 min at 4°C, the soluble supernatant was subjected to western blot analysis.

### 4.11 RNA Extraction and Real‐Time PCR Analysis

Total RNA was prepared from cells using Trizol reagent (Takara, Shiga, Japan), according to a modified protocol provided by the manufacturer. The quality of RNA was measured using NanoDrop ND‐1000 spectrophotometer. Following reverse transcription, the RNA expression levels were examined by quantitative PCR (qPCR) in triplicate on LightCycler 480 Real Time PCR system (Roche) using the SYBR Green Real‐Time PCR Master Mix (Takara, Shiga, Japan) (Table 1).

**TABLE 1:** The primers used for qPCR.

Primers	Sequence
ZIP8 forward primer	CTGTCAGAAATAGGGACGA
ZIP8 reverse primer	AAGTTGAATAGCAAGGCTT
DMT1 forward primer	TCCTCTGTGGGGTGGCGTT
DMT1 reverse (primer)	CCTGGCTCTGGCTGGGTTT
ZIP14 forward(primer) ZIP14 reverse primer	ACCCTCTACTCCAACGCCC
	CGCTGTTGAAGTGTGGGGA
Actin forward primer Actin reverse primer	GTGACAGCAGTCGGTTGGA
	AGTGGGGTGGCTTTTAGGA

### 4.12 In Vivo Tumor Model

Select C57BL/6 mice with an average age of approximately 6 weeks (purchased from the Experimental Animal Center of Southern Medical University). Initially, adequately cultured Lewis cells are processed, washed twice with cold PBS, and then suspended in physiological saline to adjust the cell density to 4 × 10^6^ cells/mL. Subsequently, 125 μL of the cell suspension is inoculated into the right abdomen of each mouse [49]. Once the tumor size reaches around 70 mm^3^, the mice are randomly assigned to groups and administered treatments: control (PBS, i.p.), DHA (20 mg/kg, i.g.), DDP (2 mg/kg, i.p.), DHA combined with DDP, and the combination with DFO (100 mg/kg, i.p.).

### 4.13 Cell Transfection

The sequences of the siRNA targeting TFRC and the shRNA targeting ZIP14 are listed in Table 2. All the siRNA and shRNA were synthesized by Tsingke Biotechnology (Tsingke Biotechnology, Beijing, China). To achieve stable knockdown of ZIP14, we utilized the pLKO.1‐puro vector system, a commonly used lentiviral vector for shRNA delivery. Following transduction with the shRNA‐expressing lentivirus, cells were selected with puromycin to establish stable knockdown lines. For transient knockdown of TFRC, we employed Lipofectamine RNAiMAX as the transfection reagent. The siRNA was transfected into the cells at a final concentration of 20 nM according to the manufacturer's instructions, and cells were incubated with the transfection mixture for 72 h to achieve effective gene silencing.

**TABLE 2:** The sequences of (siTFRC) targeting TFRC and (shZIP14) targeting ZIP14.

Name	Sequences
siTFRC	Sense: 5′‐GGAUUCCUGAGUUGAACAA‐3′
	Antisense: 5′‐UUGUUCAACUCAGGAAUCC‐3′
shZIP14	Sense: 5′‐GGAACCTCTCCACGTGCTTTA‐3′
	Antisense: 5′‐TAAAGCACGTGGAGAGGTTCC‐3′

### 4.14 Statistical Analysis

All statistical calculations were performed using GraphPad Prism (version 8.0). The quantitative data were expressed as mean ± SD or mean ± SEM. The differences between two groups were performed using the Student's *t*‐test. Comparisons among multiple groups were analyzed by one‐way ANOVA. Statistical significance was defined at *p < 0.05, **p < 0.01, and ***p < 0.001.

## Author Contributions

**Zhuoying Yang:** data curation (equal), writing – original draft (equal). **Zehao Zhou:** methodology (equal). **Qingyu Meng:** funding acquisition (equal). **Zhijie Chen:** methodology (equal). **Liang Yun:** resources (equal). **Jianjun Jiang:** software (equal). **Yujing He:** software (equal). **Meijuan Dian:** methodology (equal). **Ruihao Zhang:** software (equal). **Haotian Ge:** methodology (equal). **Tianbao Yan:** software (equal). **Biying Men:** methodology (equal). **Zichao Li:** methodology (equal). **Xu Wu:** funding acquisition (equal). **Junming He:** funding acquisition (equal), resources (equal). **Shuan Rao:** conceptualization (lead), funding acquisition (lead), writing – original draft (lead).

## Ethics Statement

All animal experimental procedures were approved by the Internal Animal Care and Use Committee at Animal Research Committee of Southern Medical University Nanfang hospital (approval numbers for animal experiments: IACUC‐LAC‐20220926‐001). The Ethics Committee and Animal Research Committee of Southern Medical University Nanfang hospital approved this study (NFEC‐2021‐250). All experiments in this study were ethically compliant. The patient‐derived lung cancer tissue samples and paraffin‐embedded tissues were obtained from the department of Thoracic in Nanfang hospital with patients' written informed consent.

## Consent

The authors have nothing to report.

## Conflicts of Interest

The authors declare no conflicts of interest.

## Supporting information


**Figure S1:** Combined treatment of DHA and DDP induce ferroptosis in lung cancer cells. (A) Gene Set Enrichment Analysis (GSEA) of the ferroptosis pathway in the combination of DHA (20 μΜ) with DDP (10 μΜ) in A549 cells. (B) Representative flow cytometry analysis of ROS levels cells (left) and ferrous iron levels (right) in A549 and H1975 cells treated with DHA (20 μΜ for A549 or 40 μΜ for H1975) and DDP (10 μΜ) alone or in combination for 48 h. (C) Representative flow cytometry analysis of ROS levels (left) and ferrous iron levels in A549 and H1975 cells treated with DHA (20 μΜ for A549 or 40 μΜ for H1975) and DDP (10 μΜ) with or without DFO (50 μΜ), cells were pretreated with DFO (50 μΜ) for 12 h. (D) Intracellular ROS levels with or without DFO (50 μΜ) in both DDP (10 μΜ) and DHA (20 μΜ for A549 or 40 μΜ for H1975)‐treated A549 or H1975 cells, mean ± SD, *n* = 3. (E) Evaluation of apoptosis in A549 cells induced by treatment with DHA (20 μM) and DDP (10 μM) alone or in combination for 48 h, using the Annexin V/PI staining assay. (F) Western blot was used to detect the expression of the autophagy indicator proteins (P62 and LC3B) in A549 cells treated with DHA (20 μΜ) and DDP (10 μΜ) alone or in combination for 48 h. (G) In vivo fluorescence imaging of the subcutaneous tumor model under mono or combined treatment with DHA (20 mg/kg) or DDP (10 mg/kg), DHA (20 mg/kg) and DDP (10 mg/kg), DHA (20 mg/kg) and DDP (10 mg/kg) and DFO (100 mg/kg). (H) Body weight of mice in (Figure S2G,H), mean ± SD, *n* = 6. ***p < 0.005.

**Figure S2:** DHA sensitizes lung cancer cells to cisplatin treatment via ZIP14. (A) Molecular docking modes of dihydroartemisinin with Transferrin receptor. The molecule in blue was dihydroartemisinin and the residues in green were donor hydrogen bonds. The yellow dashed lines represented hydrogen bonds. (B) Molecular docking of dihydroartemisinin with ferrous iron transporter ZIP14. The molecule in blue was dihydroartemisinin and the residues in green were donor hydrogen bonds. The yellow dashed lines represented hydrogen bonds. (C) CETSA‐WB determined the thermal stabilization of the ZIP14 interaction with DHA at a series of temperatures from 35°C to 60°C in A549 and H1975 cells. (D) The transcription level of ZIP14 was measured by qPCR after RNA interference using shRNA in H1975 cells, mean ± SD, *n* = 3. (E) Western blot was used to detect the expression ZIP14 proteins in shNC and shZIP14 H1975 cell. (F) Membrane‐associated ZIP14 protein levels in shNC and shZIP14 H1975 cells were measured by flow cytometry. (G) The viability of shNC and shZIP14 H1975 cells treated with DHA (40 μM) and DDP (10 μM) for 48 h by CCK‐8 assay, mean ± SD, *n* = 3. (H) Western blot was used to detect the expression of the ferroptosis‐associated proteins in shNC or shZIP14 H1975 cells treated with DHA (40 μM) and DDP (10 μM) for 48 h. (I) Flow cytometry was used to evaluate changes in ROS (left) and ferrous iron (right) levels in shNC or shZIP14 H1975 cells treated with DHA (40 μM) and DDP (10 μM) for 48 h. ***p < 0.005.

**Figure S3:** Sequential administration of DDP and DHA induces higher ZIP14 expression and more ferroptosis in lung cancer cells. (A) Quantification of the ZIP14 expression in A549 or H1975 cells treated with combination of DHA and DDP by flow cytometry in (Figure 5D). (B) The schematic diagram illustrates drug administration and experimental design. (Control group: treated with DMSO for 48 h; DHA&DDP group: treated with DHA (20 μM) and DDP (10 μM) simultaneously for 48 h; DHA‐DDP group: treated with DHA (20 μM) for 24 h then adding DDP (10 μM) for 24 h; DDP‐DHA group: treated with DDP (10 μM) for 24 h then adding DHA (20 μM) for 24 h). (C, D) Detection of ZIP14 levels in A549 or H1975 cells using flow cytometry. Cells were treated with DHA (20 μΜ for A549 or 40 μΜ for H1975) and DDP (10 μΜ) simultaneously, or treated with DHA (20 μΜ for A549 or 40 μΜ for H1975) and DDP (10 μΜ) sequentially, mean ± SD, *n* = 3. (E) The mean fluorescence intensity of intracellular ROS in A549 and H1975 cells treated with DHA (20 μΜ for A549 or 40 μΜ for H1975) and DDP (10 μΜ) simultaneously or sequentially. Mean ± SD, *n* = 3. (F) The mean fluorescence intensity of ferrous iron levels in A549 and H1975 cells treated with DHA (20 μΜ for A549 or 40 μΜ for H1975) and DDP (10 μΜ) simultaneously or sequentially. Mean ± SD, *n* = 3. *p < 0.05, **p < 0.01, ***p < 0.001, ns, statistically not significant.

## Data Availability

All data and materials generated during this study are included either in this article or in the supplementary information files.

## References

[1] A. Leiter , R. R. Veluswamy , and J. P. Wisnivesky , “The Global Burden of Lung Cancer: Current Status and Future Trends,” Nature Reviews. Clinical Oncology 20, no. 9 (2023): 624–639. 10.1038/s41571-023-00798-3 37479810

[2] Y. Li , B. Yan , and S. He , “Advances and Challenges in the Treatment of Lung Cancer,” Biomedicine & Pharmacotherapy 169 (2023): 115891. 37979378 10.1016/j.biopha.2023.115891

[3] M. Wang , R. S. Herbst , and C. Boshoff , “Toward Personalized Treatment Approaches for Non‐Small‐Cell Lung Cancer,” Nature Medicine 27, no. 8 (2021): 1345–1356. 10.1038/s41591-021-01450-2 34385702

[4] S. N. Aleksakhina , A. Kashyap , and E. N. Imyanitov , “Mechanisms of Acquired Tumor Drug Resistance,” Biochimica et Biophysica Acta 1872, no. 2 (2019): 188310. 31442474 10.1016/j.bbcan.2019.188310

[5] A. C. Gonçalves , E. Richiardone , J. Jorge , et al., “Impact of Cancer Metabolism on Therapy Resistance—Clinical Implications,” Drug Resistance Updates 59 (2021): 100797. 34955385 10.1016/j.drup.2021.100797

[6] A. Lahiri , A. Maji , P. D. Potdar , et al., “Lung Cancer Immunotherapy: Progress, Pitfalls, and Promises,” Molecular Cancer 22, no. 1 (2023): 40. 36810079 10.1186/s12943-023-01740-y PMC9942077

[7] B. Li , H. Shao , L. Gao , H. Li , H. Sheng , and L. Zhu , “Nano‐Drug Co‐Delivery System of Natural Active Ingredients and Chemotherapy Drugs for Cancer Treatment: A Reviews,” Drug Delivery 29, no. 1 (2022): 2130–2161. 35815678 10.1080/10717544.2022.2094498 PMC9275501

[8] A. F. Dos Santos , G. Fazeli , T. N. Xavier da Silva , and J. P. Friedmann Angeli , “Ferroptosis: Mechanisms and Implications for Cancer Development and Therapy Response,” Trends in Cell Biology 33, no. 12 (2023): 1062–1076. 37230924 10.1016/j.tcb.2023.04.005

[9] J. Kryczka , J. Kryczka , K. H. Czarnecka‐Chrebelska , and E. Brzeziańska‐Lasota , “Molecular Mechanisms of Chemoresistance Induced by Cisplatin in NSCLC Cancer Therapy,” International Journal of Molecular Sciences 22, no. 16 (2021): 8885. 34445588 10.3390/ijms22168885 PMC8396273

[10] X. Jiang , B. R. Stockwell , and M. Conrad , “Ferroptosis: Mechanisms, Biology and Role in Disease,” Nature Reviews. Molecular Cell Biology 22, no. 4 (2021): 266–282. 33495651 10.1038/s41580-020-00324-8 PMC8142022

[11] D. Tang , X. Chen , R. Kang , and G. Kroemer , “Ferroptosis: Molecular Mechanisms and Health Implications,” Cell Research 31, no. 2 (2021): 107–125. 33268902 10.1038/s41422-020-00441-1 PMC8026611

[12] C. Zhang , X. Liu , S. Jin , Y. Chen , and R. Guo , “Ferroptosis in Cancer Therapy: A Novel Approach to Reversing Drug Resistance,” Molecular Cancer 21, no. 1 (2022): 47. 35151318 10.1186/s12943-022-01530-y PMC8840702

[13] Y. Mou , J. Wang , J. Wu , et al., “Ferroptosis, A New Form of Cell Death: Opportunities and Challenges in Cancer,” Journal of Hematology & Oncology 12, no. 1 (2019): 34. 30925886 10.1186/s13045-019-0720-y PMC6441206

[14] M. D. Knutson , “Non‐Transferrin‐Bound Iron Transporters,” Free Radical Biology & Medicine 133 (2019): 101–111. 30316781 10.1016/j.freeradbiomed.2018.10.413

[15] R. Coffey and M. D. Knutson , “The Plasma Membrane Metal‐Ion Transporter ZIP14 Contributes to Nontransferrin‐Bound Iron Uptake by Human β‐Cells,” American Journal of Physiology. Cell Physiology 312, no. 2 (2017): C169–C175. 27903581 10.1152/ajpcell.00116.2016 PMC5336597

[16] Y. Xia , M. Sun , H. Huang , and W. L. Jin , “Drug Repurposing for Cancer Therapy,” Signal Transduction and Targeted Therapy 9, no. 1 (2024): 92. 38637540 10.1038/s41392-024-01808-1 PMC11026526

[17] F. R. Weth , G. B. Hoggarth , A. F. Weth , et al., “Unlocking Hidden Potential: Advancements, Approaches, and Obstacles in Repurposing Drugs for Cancer Therapy,” British Journal of Cancer 130, no. 5 (2024): 703–715. 38012383 10.1038/s41416-023-02502-9 PMC10912636

[18] S. Zhu , Q. Yu , C. Huo , et al., “Ferroptosis: A Novel Mechanism of Artemisinin and its Derivatives in Cancer Therapy,” Current Medicinal Chemistry 28, no. 2 (2021): 329–345, 10.2174/0929867327666200121124404. 31965935

[19] T. Efferth , “From Ancient Herb to Modern Drug: Artemisia Annua and Artemisinin for Cancer Therapy,” Seminars in Cancer Biology 46 (2017): 65–83. 28254675 10.1016/j.semcancer.2017.02.009

[20] K. H. Wong , D. Yang , S. Chen , C. He , and M. Chen , “Development of Nanoscale Drug Delivery Systems of Dihydroartemisinin for Cancer Therapy: A Review,” Asian Journal of Pharmaceutical Sciences 17, no. 4 (2022): 475–490. 36105316 10.1016/j.ajps.2022.04.005 PMC9459003

[21] J. Du , X. Wang , Y. Li , et al., “DHA Exhibits Synergistic Therapeutic Efficacy With Cisplatin to Induce Ferroptosis in Pancreatic Ductal Adenocarcinoma via Modulation of Iron Metabolism,” Cell Death & Disease 12, no. 7 (2021): 705. 34262021 10.1038/s41419-021-03996-y PMC8280115

[22] Z. Gao , Y. Li , C. You , et al., “Iron Oxide Nanocarrier‐Mediated Combination Therapy of Cisplatin and Artemisinin for Combating Drug Resistance Through Highly Increased Toxic Reactive Oxygen Species Generation,” ACS Applied Bio Materials 1, no. 2 (2018): 270–280. 10.1021/acsabm.8b00056 35016370

[23] W. Li , G. Ma , Y. Deng , Q. Wu , Z. Wang , and Q. Zhou , “Artesunate Exhibits Synergistic Anti‐Cancer Effects With Cisplatin on Lung Cancer A549 Cells by Inhibiting MAPK Pathway,” Gene 766 (2021): 145134. 32898605 10.1016/j.gene.2020.145134

[24] A. Ianevski , A. K. Giri , and T. Aittokallio , “SynergyFinder 3.0: An Interactive Analysis and Consensus Interpretation of Multi‐Drug Synergies Across Multiple Samples,” Nucleic Acids Research 50, no. W1 (2022): W739–W743. 35580060 10.1093/nar/gkac382 PMC9252834

[25] M. Fransolet , L. Henry , S. Labied , A. Noël , M. Nisolle , and C. Munaut , “In Vitro Evaluation of the Anti‐Apoptotic Drug Z‐VAD‐FMK on Human Ovarian Granulosa Cell Lines for Further Use in Ovarian Tissue Transplantation,” Journal of Assisted Reproduction and Genetics 32, no. 10 (2015): 1551–1559. 26169075 10.1007/s10815-015-0536-9 PMC4615917

[26] T. M. K. Motawi , Z. M. Abdel‐Nasser , and N. N. Shahin , “Ameliorative Effect of Necrosulfonamide in a Rat Model of Alzheimer's Disease: Targeting Mixed Lineage Kinase Domain‐Like Protein‐Mediated Necroptosis,” ACS Chemical Neuroscience 11, no. 20 (2020): 3386–3397. 32936609 10.1021/acschemneuro.0c00516

[27] P. Li , M. Jiang , K. Li , et al., “Glutathione Peroxidase 4‐Regulated Neutrophil Ferroptosis Induces Systemic Autoimmunity,” Nature Immunology 22, no. 9 (2021): 1107–1117. 34385713 10.1038/s41590-021-00993-3 PMC8609402

[28] L. Rochette , G. Dogon , E. Rigal , M. Zeller , Y. Cottin , and C. Vergely , “Lipid Peroxidation and Iron Metabolism: Two Corner Stones in the Homeostasis Control of Ferroptosis,” International Journal of Molecular Sciences 24, no. 1 (2022): 449. 36613888 10.3390/ijms24010449 PMC9820499

[29] S. Li , F. Li , L. Xu , et al., “TLR2 Agonist Promotes Myeloid‐Derived Suppressor Cell Polarization via Runx1 in Hepatocellular Carcinoma,” International Immunopharmacology 111 (2022): 109168. 35998504 10.1016/j.intimp.2022.109168

[30] H. Wu , K. Gong , Y. Qin , et al., “In Silico Analysis of the Potential Mechanism of a Preventive Chinese Medicine Formula on Coronavirus Disease 2019,” Journal of Ethnopharmacology 275 (2021): 114098. 33831468 10.1016/j.jep.2021.114098 PMC8020622

[31] S. Forli , R. Huey , M. E. Pique , M. F. Sanner , D. S. Goodsell , and A. J. Olson , “Computational Protein‐Ligand Docking and Virtual Drug Screening With the AutoDock Suite,” Nature Protocols 11, no. 5 (2016): 905–919. 27077332 10.1038/nprot.2016.051 PMC4868550

[32] J. Lee and D. H. Hyun , “The Interplay Between Intracellular Iron Homeostasis and Neuroinflammation in Neurodegenerative Diseases,” Antioxidants 12, no. 4 (2023): 918. 37107292 10.3390/antiox12040918 PMC10135822

[33] L. Zhou , J. Jiang , Z. Huang , et al., “Hypoxia‐Induced lncRNA STEAP3‐AS1 Activates Wnt/β‐Catenin Signaling to Promote Colorectal Cancer Progression by Preventing m6A‐Mediated Degradation of STEAP3 mRNA,” Molecular Cancer 21, no. 1 (2022): 168. 35986274 10.1186/s12943-022-01638-1 PMC9392287

[34] J. W. W. Winslow , K. H. Limesand , and N. Zhao , “The Functions of ZIP8, ZIP14, and ZnT10 in the Regulation of Systemic Manganese Homeostasis,” International Journal of Molecular Sciences 21, no. 9 (2020): 3304. 32392784 10.3390/ijms21093304 PMC7246657

[35] J. E. Chaft , Y. Shyr , B. Sepesi , and P. M. Forde , “Preoperative and Postoperative Systemic Therapy for Operable Non‐Small‐Cell Lung Cancer,” Journal of Clinical Oncology 40, no. 6 (2022): 546–555. 34985966 10.1200/JCO.21.01589 PMC8853628

[36] M. Yuan , L. L. Huang , J. H. Chen , J. Wu , and Q. Xu , “The Emerging Treatment Landscape of Targeted Therapy in Non‐small‐Cell Lung Cancer,” Signal Transduction and Targeted Therapy 4 (2019): 61. 31871778 10.1038/s41392-019-0099-9 PMC6914774

[37] M. Goldstein and M. B. Kastan , “The DNA Damage Response: Implications for Tumor Responses to Radiation and Chemotherapy,” Annual Review of Medicine 66 (2015): 129–143. 10.1146/annurev-med-081313-121208 25423595

[38] M. Yu , B. Qi , W. Xiaoxiang , J. Xu , and X. Liu , “Baicalein Increases Cisplatin Sensitivity of A549 Lung Adenocarcinoma Cells via PI3K/Akt/NF‐κB Pathway,” Biomedicine & Pharmacotherapy 90 (2017): 677–685. 28415048 10.1016/j.biopha.2017.04.001

[39] X. L. Su , J. W. Wang , H. Che , et al., “Clinical Application and Mechanism of Traditional Chinese Medicine in Treatment of Lung Cancer,” Chinese Medical Journal 133, no. 24 (2020): 2987–2997. 33065603 10.1097/CM9.0000000000001141 PMC7752681

[40] T. Tadokoro , M. Ikeda , T. Ide , et al., “Mitochondria‐Dependent Ferroptosis Plays a Pivotal Role in Doxorubicin Cardiotoxicity,” JCI Insight 8, no. 6 (2023): e169756. 36946465 10.1172/jci.insight.169756 PMC10070098

[41] F. Cheng , J. Dou , Y. Yang , et al., “Drug‐Induced Lactate Confers Ferroptosis Resistance via p38‐SGK1‐NEDD4L‐Dependent Upregulation of GPX4 in NSCLC Cells,” Cell Death Discovery 9, no. 1 (2023): 165. 37188685 10.1038/s41420-023-01463-5 PMC10185500

[42] H. Li , K. Yu , H. Hu , et al., “METTL17 Coordinates Ferroptosis and Tumorigenesis by Regulating Mitochondrial Translation in Colorectal Cancer,” Redox Biology 71 (2024): 103087. 38377789 10.1016/j.redox.2024.103087 PMC10884776

[43] Y. M. Shyu , L. Y. Liu , and Y. J. Chuang , “Synergistic Effect of Simultaneous Versus Sequential Combined Treatment of Histone Deacetylase Inhibitor Valproic Acid With Etoposide on Melanoma Cells,” International Journal of Molecular Sciences 22, no. 18 (2021): 10029. 34576202 10.3390/ijms221810029 PMC8467070

[44] W. Huang , Y. Wang , T. He , et al., “Arteannuin B Enhances the Effectiveness of Cisplatin in Non‐Small Cell Lung Cancer by Regulating Connexin 43 and MAPK Pathway,” American Journal of Chinese Medicine 50, no. 7 (2022): 1963–1992. 36040035 10.1142/S0192415X22500847

[45] F. Zhao , O. Vakhrusheva , S. D. Markowitsch , et al., “Artesunate Impairs Growth in Cisplatin‐Resistant Bladder Cancer Cells by Cell Cycle Arrest, Apoptosis and Autophagy Induction,” Cells 9, no. 12 (2020): 2643. 33316936 10.3390/cells9122643 PMC7763932

[46] P. Lv , S. Man , L. Xie , L. Ma , and W. Gao , “Pathogenesis and Therapeutic Strategy in Platinum Resistance Lung Cancer,” Biochimica et Biophysica Acta 1876, no. 1 (2021): 188577. 34098035 10.1016/j.bbcan.2021.188577

[47] Y. Q. Qu , L. L. Song , S. W. Xu , et al., “Pomiferin Targets SERCA, mTOR, and P‐Gp to Induce Autophagic Cell Death in Apoptosis‐Resistant Cancer Cells, and Reverses the MDR Phenotype in Cisplatin‐Resistant Tumors In Vivo,” Pharmacological Research 191 (2023): 106769. 37061145 10.1016/j.phrs.2023.106769

[48] M. Kim , H. Mun , C. O. Sung , et al., “Patient‐Derived Lung Cancer Organoids as In Vitro Cancer Models for Therapeutic Screening,” Nature Communications 10, no. 1 (2019): 3991. 10.1038/s41467-019-11867-6 PMC6728380 31488816

[49] T. Shirai , N. Tsukiji , T. Sasaki , et al., “Cancer‐Associated Fibroblasts Promote Venous Thrombosis Through Podoplanin/CLEC‐2 Interaction in Podoplanin‐Negative Lung Cancer Mouse Model,” Journal of Thrombosis and Haemostasis 21, no. 11 (2023): 3153–3165. 37473844 10.1016/j.jtha.2023.07.005

