# Supplementary figures and images for: Dihydroartemisinin Sensitizes Lung Cancer Cells to Cisplatin Treatment by Upregulating ZIP14 Expression and Inducing Ferroptosis
Source: Cancer Med. 2024 Oct 12;13(19):e70271. doi: 10.1002/cam4.70271 (PMC11470233; doi:10.1002/cam4.70271)

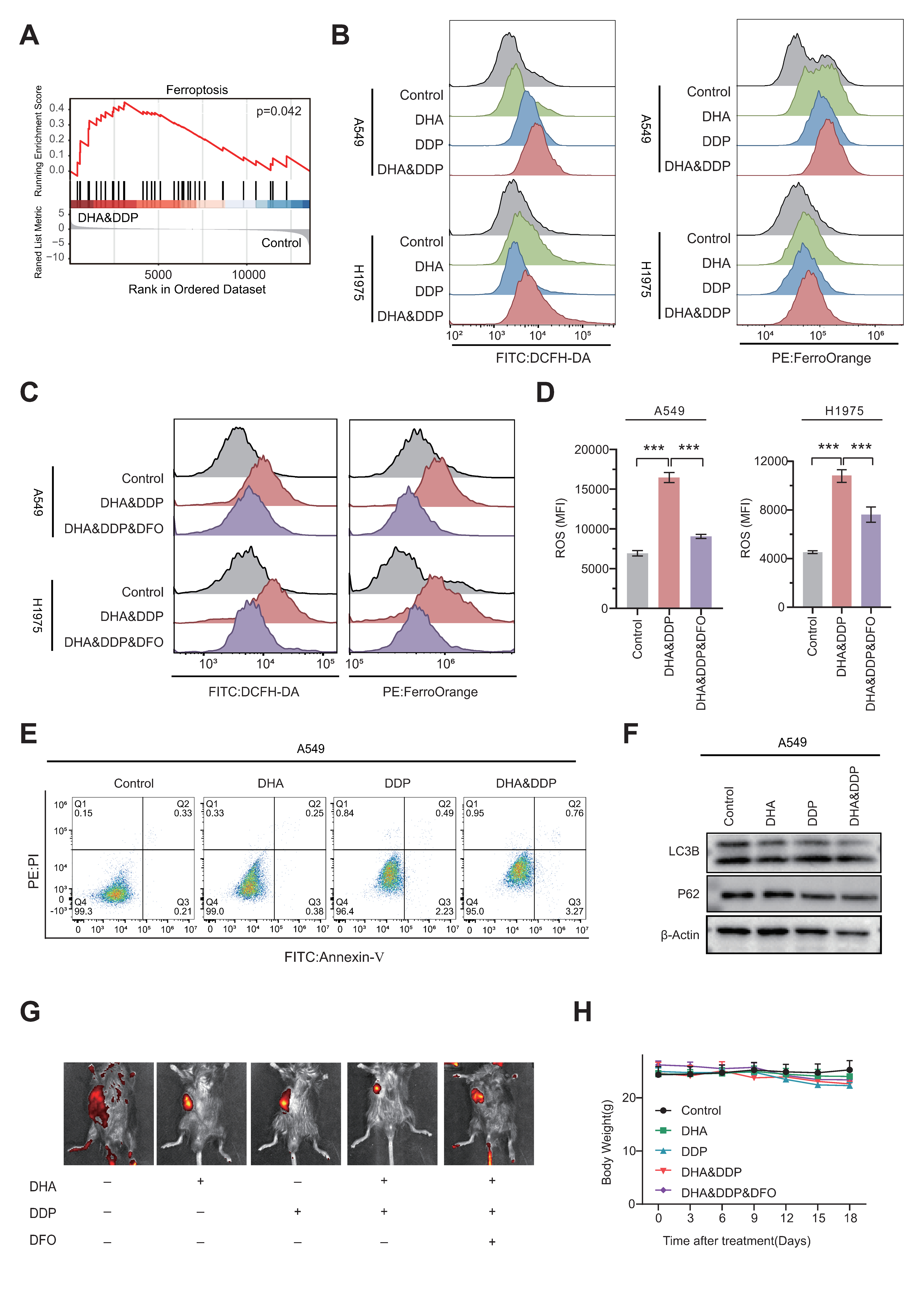

Supplement: Supplementary file 1 — Figure S1: Combined treatment of DHA and DDP induce ferroptosis in lung cancer cells. (A) Gene Set Enrichment Analysis (GSEA) of the ferroptosis pathway in the combination of DHA (20 μΜ) with DDP (10 μΜ) in A549 cells. (B) Representative flow cytometry analysis of ROS levels cells (left) and ferrous iron levels (right) in A549 and H1975 cells treated with DHA (20 μΜ for A549 or 40 μΜ for H1975) and DDP (10 μΜ) alone or in combination for 48 h. (C) Representative flow cytometry analysis of ROS levels (left) and ferrous iron levels in A549 and H1975 cells treated with DHA (20 μΜ for A549 or 40 μΜ for H1975) and DDP (10 μΜ) with or without DFO (50 μΜ), cells were pretreated with DFO (50 μΜ) for 12 h. (D) Intracellular ROS levels with or without DFO (50 μΜ) in both DDP (10 μΜ) and DHA (20 μΜ for A549 or 40 μΜ for H1975)‐treated A549 or H1975 cells, mean ± SD, n = 3. (E) Evaluation of apoptosis in A549 cells induced by treatment with DHA (20 μM) and DDP (10 μM) alone or in combination for 48 h, using the Annexin V/PI staining assay. (F) Western blot was used to detect the expression of the autophagy indicator proteins (P62 and LC3B) in A549 cells treated with DHA (20 μΜ) and DDP (10 μΜ) alone or in combination for 48 h. (G) In vivo fluorescence imaging of the subcutaneous tumor model under mono or combined treatment with DHA (20 mg/kg) or DDP (10 mg/kg), DHA (20 mg/kg) and DDP (10 mg/kg), DHA (20 mg/kg) and DDP (10 mg/kg) and DFO (100 mg/kg). (H) Body weight of mice in (Figure S2G,H), mean ± SD, n = 6. ***p < 0.005. [file CAM4-13-e70271-s001.jpg]

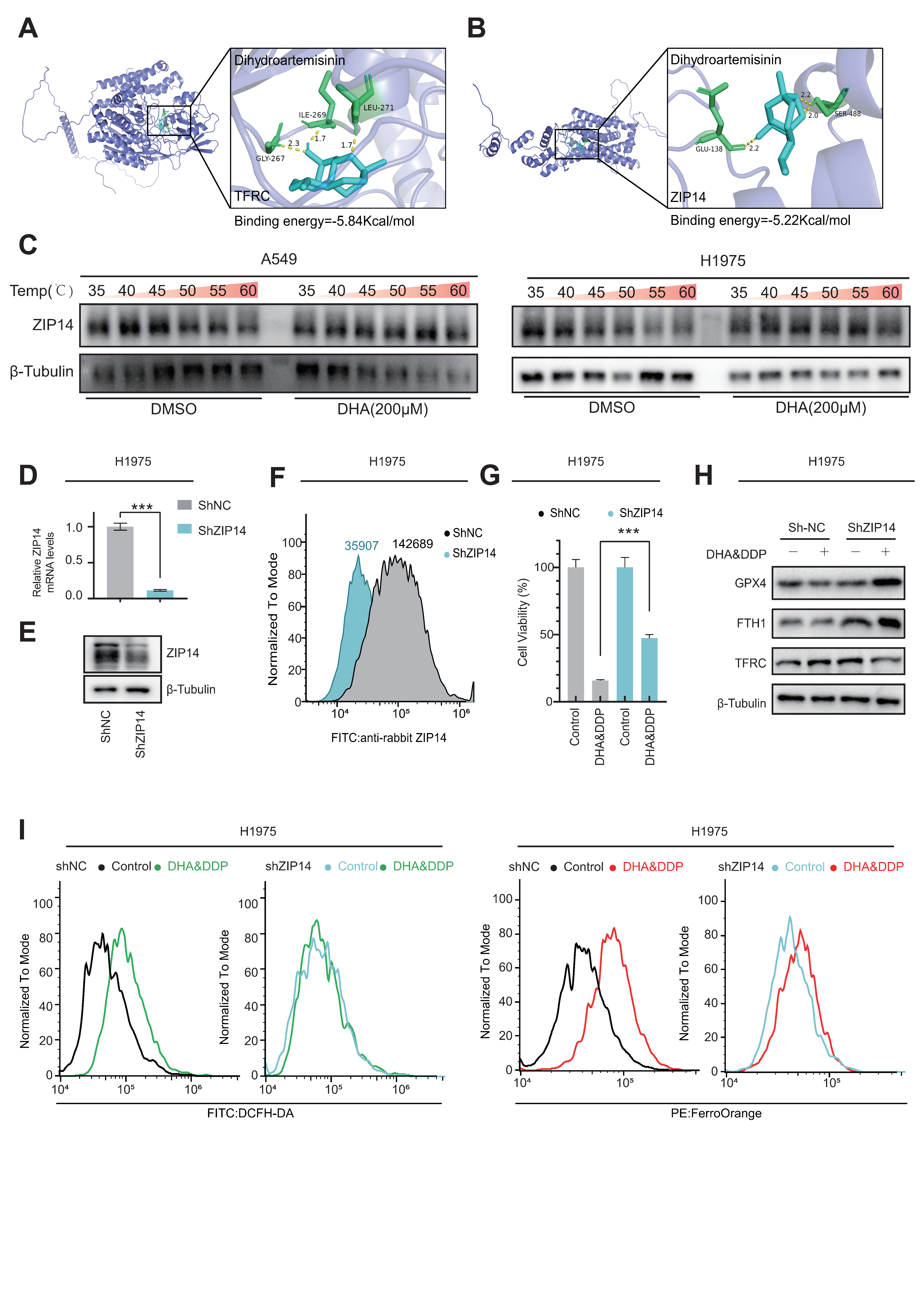

Supplement: Supplementary file 2 — Figure S2: DHA sensitizes lung cancer cells to cisplatin treatment via ZIP14. (A) Molecular docking modes of dihydroartemisinin with Transferrin receptor. The molecule in blue was dihydroartemisinin and the residues in green were donor hydrogen bonds. The yellow dashed lines represented hydrogen bonds. (B) Molecular docking of dihydroartemisinin with ferrous iron transporter ZIP14. The molecule in blue was dihydroartemisinin and the residues in green were donor hydrogen bonds. The yellow dashed lines represented hydrogen bonds. (C) CETSA‐WB determined the thermal stabilization of the ZIP14 interaction with DHA at a series of temperatures from 35°C to 60°C in A549 and H1975 cells. (D) The transcription level of ZIP14 was measured by qPCR after RNA interference using shRNA in H1975 cells, mean ± SD, n = 3. (E) Western blot was used to detect the expression ZIP14 proteins in shNC and shZIP14 H1975 cell. (F) Membrane‐associated ZIP14 protein levels in shNC and shZIP14 H1975 cells were measured by flow cytometry. (G) The viability of shNC and shZIP14 H1975 cells treated with DHA (40 μM) and DDP (10 μM) for 48 h by CCK‐8 assay, mean ± SD, n = 3. (H) Western blot was used to detect the expression of the ferroptosis‐associated proteins in shNC or shZIP14 H1975 cells treated with DHA (40 μM) and DDP (10 μM) for 48 h. (I) Flow cytometry was used to evaluate changes in ROS (left) and ferrous iron (right) levels in shNC or shZIP14 H1975 cells treated with DHA (40 μM) and DDP (10 μM) for 48 h. ***p < 0.005. [file CAM4-13-e70271-s002.jpg]

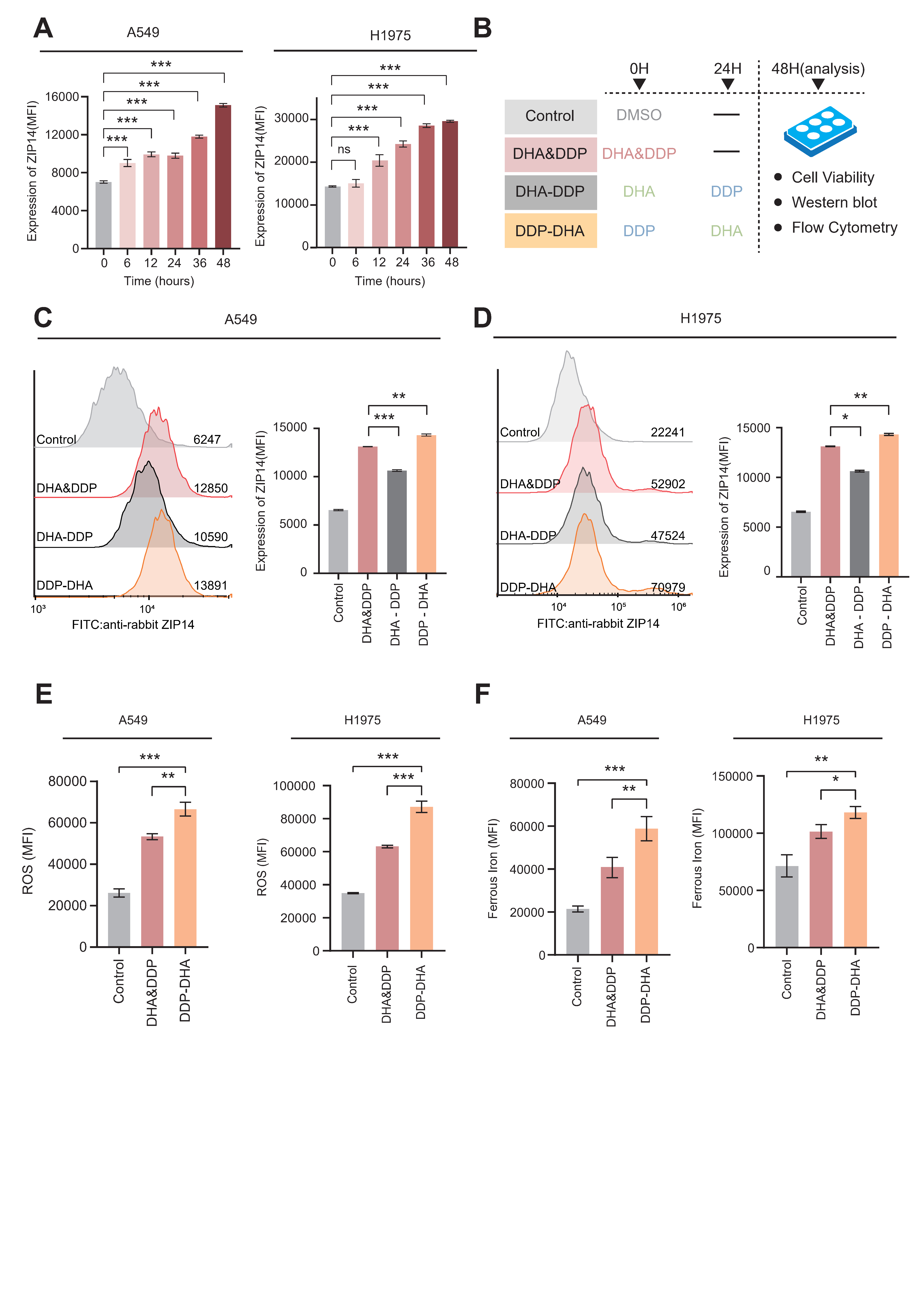

Supplement: Supplementary file 3 — Figure S3: Sequential administration of DDP and DHA induces higher ZIP14 expression and more ferroptosis in lung cancer cells. (A) Quantification of the ZIP14 expression in A549 or H1975 cells treated with combination of DHA and DDP by flow cytometry in (Figure 5D). (B) The schematic diagram illustrates drug administration and experimental design. (Control group: treated with DMSO for 48 h; DHA&DDP group: treated with DHA (20 μM) and DDP (10 μM) simultaneously for 48 h; DHA‐DDP group: treated with DHA (20 μM) for 24 h then adding DDP (10 μM) for 24 h; DDP‐DHA group: treated with DDP (10 μM) for 24 h then adding DHA (20 μM) for 24 h). (C, D) Detection of ZIP14 levels in A549 or H1975 cells using flow cytometry. Cells were treated with DHA (20 μΜ for A549 or 40 μΜ for H1975) and DDP (10 μΜ) simultaneously, or treated with DHA (20 μΜ for A549 or 40 μΜ for H1975) and DDP (10 μΜ) sequentially, mean ± SD, n = 3. (E) The mean fluorescence intensity of intracellular ROS in A549 and H1975 cells treated with DHA (20 μΜ for A549 or 40 μΜ for H1975) and DDP (10 μΜ) simultaneously or sequentially. Mean ± SD, n = 3. (F) The mean fluorescence intensity of ferrous iron levels in A549 and H1975 cells treated with DHA (20 μΜ for A549 or 40 μΜ for H1975) and DDP (10 μΜ) simultaneously or sequentially. Mean ± SD, n = 3. *p < 0.05, **p < 0.01, ***p < 0.001, ns, statistically not significant. [file CAM4-13-e70271-s003.jpg]
